# CAREGIVER PARTICIPATION IN VIDEO VISITS: CHALLENGE OR OPPORTUNITY?

**DOI:** 10.1093/geroni/igac059.019

**Published:** 2022-12-20

**Authors:** Megan Gately, Lauren Moo

## Abstract

Rapid expansion of telehealth in response to COVID revealed a digital divide for many patients, particularly older adults. Given the technical complexity of video visits (which may include downloading novel software and enabling a camera and microphone), video visits may be out of reach for older patients with less technological experience or with age and condition-related changes such as sensory loss or cognitive impairment. Involving caregivers in video visits (particularly technical set-up) may not only increase patient access but also enhance clinical care by allowing for collaboration with family. Caregivers may themselves benefit from video visits, given that video offers increased options for caregiver support. Though caregivers are often identified as critical components to older adults’ accessing telehealth and may also benefit from telehealth services, caregivers’ own technical needs are not well-understood. This symposium discusses caregivers’ involvement in telehealth from multiple perspectives. The first presentation includes findings from a national clinician survey about caregivers’ support role in occupational therapy video visits, including barriers and benefits (Gately et al). The second presentation includes findings from a regional survey of interprofessional clinicians about telehealth modalities to provide dementia family caregiver support during COVID-19 (Quach et al). The third presentation includes family caregivers’ technology assistance requirements before and during a virtual, seven-session group skills training program, including benefits of individualized assistance (Moo et al). The fourth presentation includes caregiver perspectives about tele-geriatrics visits, highlighting caregivers’ support role and enhancements of video versus phone (Boudreau et al).

